# Optimization and Identification of Single Mutation in Hemoglobin Variants with 2,2,2 Trifluoroethanol Modified Digestion Method and Nano−LC Coupled MALDI MS/MS

**DOI:** 10.3390/molecules27196357

**Published:** 2022-09-26

**Authors:** Pushpanjali Dasauni, Nirpendra Singh, Varun Chhabra, Manoranjan Mahapatra, Renu Saxena, Suman Kundu

**Affiliations:** 1 Department of Biochemistry, University of Delhi South Campus, New Delhi 110021, India; 2 Institute for Stem Cell Science & Regenerative Medicine (inStem), Bangalore 560065, India; 3 Department of Haematology, All India Institute of Medical Sciences, New Delhi 110029, India; 4 Department of Path and Lab Medicine, Department of Hematopathology, Medanta, The Medicity Hospital, Gurgaon 122018, India; 5 Department of Biological Sciences, Birla Institute of Technology and Science Pilani, K.K. Birla Goa Campus, Goa 403726, India

**Keywords:** hemoglobin disorders, mass spectrometry, hemoglobin variants, liquid chromatography, MALDI TOF/TOF, nano LC−MALDI MS/MS, Hb variant identification

## Abstract

**Background:** Hemoglobin (Hb) variants arise due to point mutations in globin chains and their pathological treatments rely heavily on the identification of the nature and location of the mutation in the globin chains. Traditional methods for diagnosis such as HPLC and electrophoresis have their own limitations. Therefore, the present study aims to develop and optimize a specific method of sample processing that could lead to improved sequence coverage and analysis of Hb variants by nano LC−MALDI MS/MS. **Methods:** In our study, we primarily standardized various sample processing methods such as conventional digestion with trypsin followed by 10% acetonitrile treatment, digestion with multiple proteases like trypsin, Glu−C, Lys−C, and trypsin digestion subsequent to 2,2,2 trifluoroethanol (TFE) treatment. Finally, the peptides were identified by LC−MALDI MS/MS. All of these sample processing steps were primarily tested with recombinant Hb samples. After initial optimization, we found that the TFE method was the most suitable one and the efficiency of this method was applied in Hb variant identification based on high sequence coverage. **Results:** We developed and optimized a method using an organic solvent TFE and heat denaturation prior to digestion, resulting in 100% sequence coverage in the β−chains and 95% sequence coverage in the α−chains, which further helped in the identification of Hb mutations. A Hb variant protein sequence database was created to specify the search and reduce the search time. **Conclusion:** All of the mutations were identified using a bottom−up non−target approach. Therefore, a sensitive, robust and reproducible method was developed to identify single substitution mutations in the Hb variants from the sequence of the entire globin chains. **Biological Significance:** Over 330,000 infants are born annually with hemoglobinopathies and it is the major cause of morbidity and mortality in early childhood. Hb variants generally arise due to point mutation in the globin chains. There is high sequence homology between normal Hb and Hb variant chains. Due to this high homology between the two forms, identification of variants by mass spectrometry is very difficult and requires the full sequence coverage of α− and β−chains. As such, there is a need for a suitable method that provides 100% sequence coverage of globin chains for variant analysis by mass spectrometry. Our study provides a simple, robust, and reproducible method that is suitable for LC−MALDI and provides nearly complete sequence coverage in the globin chains. This method may be used in the near future in routine diagnosis for Hb variant analysis.

## 1. Introduction

Hemoglobin (Hb) is a polypeptide tetramer of molecular weight 64 KDa, which transports oxygen in the body. Adult Hb consists of 97% Hb A (α_2_β_2_), 2.5% Hb A_2_ (α_2_δ_2_), and up to 0.5% Hb F (α_2_γ_2_). The alpha globin gene is located on chromosome 16, while the beta, delta, and gamma globin genes are located on chromosome 11. The alpha chain contains 141 amino acid residues while the beta chain contains 146 amino acid residues. Hb disorders are caused by or are due to substitutions/insertions/ deletions in the globin chains of Hbs. In many Hb disorders, the Hb molecule gets altered structurally and functionally. In the case of some Hb disorders, the oxygen binding affinity of Hb also gets altered, which may cause various complications like anemia and, if untreated, sometimes they may become life−threatening. Hemoglobinopathies are one of the major public health problems in India [1,2] and constitute an increasing global health burden [3,4]. The treatments rely only on the successful diagnosis of Hb variants [5,6]. The routine/conventional techniques used for the identification of Hb variants have their own limitations such as the co-migration of variants in electrophoresis and the co−elution in HPLC [7,8,9]. Moreover, these techniques can diagnose variants that are already known, or at best can provide a qualitative indication of the presence of a variant. These tools can identify the presence of a variant qualitatively, but cannot identify the identity of the variant in the absence of a prior reference. None of the tools can identify the precise location of the mutation in Hb chains or the nature of the mutation.

To overcome such problems, mass spectrometry (MS) was explored as a potential tool to identify new mutations as well as to pinpoint the location and the nature of the mutation. MS emerged as a powerful diagnostic tool in the identification of Hb variants in the last three decades [10,11,12]. Nano LC coupled with the MALDI MS/MS integrated approach demonstrated better sequence coverage, which facilitated variant identification [13]. The LC−MS/MS approach is thus extremely helpful in the case of Hb variant analysis; it is generally used to separate each peptide and fragment to get improved sequence coverage with high confidence [14].

Various modifications in the protein digestion protocol for improved sequence coverage were used by different groups, including temperature, denaturants, solvents, and different proteases [15]. For improved protein digestion, the use of denaturants like 8 M urea, 6 M GuHCl, 0.11 *w*/*v* SDS, and 50% *v*/*v* TFE was mentioned in the literature with and without heat treatment [16]. 2,2,2 Trifluoroethanol (TFE) is an organic co−solvent that is known to improve protein solubility and increases denaturation in a wide range of proteins [16,17]. Using TFE instead of using other denaturants/detergents may help in preventing the sample clean−up step during the analysis of peptides. A proper sample processing protocol needs to be developed along with a high−resolution separation protocol for methods to overcome the loss of peptides before MS analysis. Trypsin digestion is typically performed with aqueous solvents, but using 80% acetonitrile resulted in digestion with higher efficiency, thus resulting in the identification of more peptides [18]. The use of 40% ACN was studied and was found to improve sequence coverage [19]. Rapigest was generally used at 0.2% concentration and is documented to support 100% trypsin activity [12]. Microwave−assisted digestion for a hemoglobin HbS sample at 150 °C for 10 min in the presence of 0.05% formic acid yielded a sequence coverage of 98.58% by MALDI TOF MS for the α−chain and 79.45% for the β−chain [20]. The HbS signature peptide was also identified with this method. Hence, there is scope for further improvement to result in 100% sequence coverage. In addition, to date, there is no dedicated protein sequence database available to search for the Hb variants after MS processing, which also limits method development.

Therefore, the aim of the present study is the optimization of modified sample processing methods used for MS to improve sequence coverage up to nearly 100% and the identification of single mutations in Hb variants from patient samples. This method may be used in the future to specifically identify the single amino acid substitution mutation in Hb disorders through a database search.

## 2. Materials and Methods

### 2.1. Materials

Acetonitrile (ACN), 2,2,2−trifluoroethanol (TFE), and water were obtained from Fluka (St. Louis, MO, USA). Ammonium bicarbonate (ABC) and Formic acid (FA) were purchased from Sigma−Aldrich (St. Louis, MO, USA). α−Cyano−4−hydroxy−cinnamic acid (α−CHCA) and sinapinic acid (SA) were obtained from Applied Biosystems (Foster City, CA, USA)**.** The enzyme “Trypsin Gold” was purchased from Promega (Madison, WI, USA) and Lys−C Endoproteinase, Glu−C Endoproteinase (MS grade) was from Thermoscientific Pierce (Rockford, IL, USA). A Hemoglobind matrix was procured from Biotech Support Group, LLC (Monmouth Junction, NJ, USA). This matrix binds hemoglobin specifically from whole blood lysate/RBC lysates.

### 2.2. Experimental Design of the Study

The study was carried out in two phases: In **Phase I**: initial screening and optimization of sample processing methods were carried out in recombination hemoglobin (rHb) samples with various methods such as 10% acetonitrile treatment, multiple proteases like trypsin, Glu−C, Lys−C, and others In **Phase II**: the most successful sample processing method i.e., the TFE treatment method, was applied for Hb variant identification.

### 2.3. Phase I: Strategies Used for Screening and Optimization of Sample Processing Methods for Complete Sequence Coverage in Recombinant Hemoglobins

#### 2.3.1. Recombinant Hemoglobin

Recombinant Hb (rHb) wild−type protein (for control) and mutants were a kind gift from Prof. John Olson, Rice University, USA and were pure with up to 90% homogeneity. The rHb samples used in the study include: rHb control, Mutant 1: rHb beta 63 (E7) His > Gly, Mutant 2: rHb beta 28 (B10) Leu > Phe, Mutant 3: Beta 67 (E11) Val > Ala, Mutant 4: Alpha 62 (E11) Val > Asn. The rHbs were used as proof of concept for optimization to obtain full sequence coverage and identification of mutation by mass spectrometry.

#### 2.3.2. Conventional Digestion of rHb

1 µL (10 µg/µL) of rHb proteins (rHb control, rhb mutant 1, and mutant 2) were taken in a microcentrifuge tube. Subsequently, 5 µL of 500 mM ammonium bicarbonate buffer (ABC buffer) of pH 8.0 was added. The sample was diluted with 43 µL of LC−MS grade water to make the final volume 50 µL. The reduction and alkylation steps were avoided since, although Hb has six cysteine side chains, there are no disulfide bonds [21]. Subsequently, 1:50 of the trypsin to sample ratio of the enzyme was taken and kept at 37 °C overnight (16 h). The reaction was stopped by formic acid (FA) to make the final concentration 0.1% of FA.

#### 2.3.3. 10% ACN Treatment Prior Trypsin Digestion

1 µL (10 µg/µL) of protein was taken in a microcentrifuge tube. The sample was diluted in 5 µL of 500 mM ABC buffer. Prior to trypsin addition, 5 µL of ACN was added to give a final ACN concentration of 5% *v*/*v* during digestion. The sample volume was finally adjusted to 50 µL by adding 38 µL of LC−MS grade water. The trypsin to sample ratio was 1:50 and kept at 37 °C overnight. The reaction was stopped by FA to make the final concentration 0.1% of FA.

#### 2.3.4. Digestion of rHbs by Various Proteases like Trypsin, Glu−C and Lys−C

Other than trypsin, Glu−C and Lys−C proteases were also used for the digestion purpose to get a high sequence coverage resulting in variant identification. 1 μL (10 μg/μL) of protein was taken in a microcentrifuge tube. 5 μL of 500 mM ABC buffer was added to maintain pH 8.0 and 5 μL of ACN to facilitate solubility of the protein. 38 μL of LC−MS grade water was added to adjust the reaction volume to 50 μL. A 1:20 enzyme−to−protein sample ratio was maintained in the reaction mixture. The tube was incubated at 37 °C for 16 hrs. The reaction was stopped by formic acid to make the final concentration 0.1% of FA.

#### 2.3.5. TFE Treatment for rHbs

1 µL (10 µg/µL) of rHb protein was taken in a microcentrifuge tube and mixed with an equal volume of water. Next, 2 µL of TFE was added to the sample (50% *v*/*v*). Samples were heated at 95 °C for 20 min, and 5 μL of 500 mM ABC buffer was added. Subsequently, 39 μL of LC−MS grade water was added to make the final concentration of TFE 5% (*v*/*v*) to maintain the activity of trypsin. Trypsin was added in a 1:50 ratio to the sample in each sample. The reaction was kept at 37 °C for approximately 16 hrs for protein digestion. Finally, the reaction was stopped by adding 0.1% FA to the solution.

### 2.4. Phase II: Identification of Hb Variants by TFE Method

#### 2.4.1. Ethical Approval and Blood Sample Collection

Ethics approval for the study was obtained from the University of Delhi South Campus (UDSC) (3/IEC/SK/Biochem/UDSC/18.02.2014) and the All India Institute of Medical Sciences (AIIMS) (IEC/OP−4/01.05.2014). Written consents were obtained from the volunteers before blood collection. The exclusion criterion for blood sample collection was people suffering from prolonged illnesses like malaria, typhoid, jaundice, etc., those receiving antibiotics and unwilling individuals. Blood samples from healthy individuals were collected at UDSC and patients suffering from Hb disorders were collected at AIIMS. The blood sample of patients suffering from disease Hb variants HbS (n = 10), HbE (n = 15) and HbD (n = 3) were collected. Blood of healthy subjects (n = 40) serving as a control was also collected. In our study, only single mutations in Hb variants were focused. Venous blood samples (3 mL) were collected in EDTA vials and kept at 4 °C until the completion of the processing step. After processing, the samples were stored at −20 °C until further analysis.

#### 2.4.2. Sample Processing and Isolation of Pure Hemoglobin

Whole blood samples were centrifuged at 400× *g* for 10 min to separate plasma which was stored in separate vials. The red blood cells (RBCs) were washed thrice in 0.9% saline to remove the buffy coat and to pack RBCs. Packed RBCs were added with a double volume of distilled water and centrifuged at 20,000× *g* for 30 min. This pellets all the cell stroma and provides the hemolysate as supernatant [22]. Pure Hb was isolated from hemolysate by using the Hemoglobind matrix, as several groups have used this matrix for the removal of Hb from composite samples [23,24,25,26]. The hemolysate was added to an equal volume of Hemoglobind matrix and mixed properly and centrifuged at 400× *g* for 2 min to pellet down the Hb bound to the Hemoglobind matrix while unbound Hb was washed away. The Hb bound to the matrix was eluted by increasing the pH of the buffer using 100 mM Tris borate, pH 9. This allows for easy, one−step isolation of Hb from hemolysate.

#### 2.4.3. Assessment of Quality of Pure Hb by SDS−PAGE, Absorption Spectroscopy and UPLC

The purity and quality of Hbs isolated from Hemoglobind were assessed using the SDS−PAGE, absorbance, and UPLC techniques. In the absorbance method, protein samples showing a protein ratio A_405nm_/A_280_ above 3.5 were considered pure, which was further confirmed by SDS−PAGE gel. Further confirmation of pure Hb is done by the running of the samples on UPLC to separate the components of Hb i.e., heme, alpha, and the beta chain. The HPLC method as described by Wajcman was converted to the UPLC method with a run time of 25 min [27]. The method involved two solvents; solvent A containing 35% acetonitrile (ACN) and 0.1% trifluoroacetic acid (TFA) in water and solvent B containing 45% ACN and 0.1% TFA in water. Buffer A and B at different gradients allowed for elution and were passed with a flow rate of 0.25 mL/min through the UPLC BEH C18 column. Hb samples were diluted in buffer A to make a solution of 10 μg/μL and 5 μL of the sample was loaded onto the column. Initially, solvent B was passed at 0%, then at 1.67 min the column was equilibrated with 35% B; at 15.67 min the solvent B was passed in a linear gradient of 35% to 55%, at 21.67 min solvent B was passed in a linear gradient of 55% to 90%, at 22.67 min it was kept same for cleaning of the column, at 23.67 min the solvent B was passed 10% and the same was true at 24.67 min. The spectra of Hb were observed at 210 and 280 nm. Peak integration and extraction were done using Empower 2.0 software (Waters, Milford, MA, USA).

#### 2.4.4. TFE Treatment of Human Hb Variant Samples

TFE treated Human Hb variants were processed similarly like TFE treated recombinant Hbs. For a detailed procedure please refer to Section 2.3.5.

### 2.5. Nano−liquid Chromatography for Separation of Peptides

For the nano−LC separation of peptides, 5 µL of in−solution digested peptides were mixed with 9 µL of 98% water, 2% ACN, and 0.1% TFA. An aliquot of 12 µL of the digested peptide was separated on a Chromolith CapRod Monolithic capillary column (150 mm × 0.1 mm RP−18 end−capped) from Merck KGaA, Darmstadt, Germany. Simultaneously, the eluted peptide was spotted on the LC−MALDI plate at an interval of 7 s after mixing with α−cyano−4−hydroxycinnamic acid matrix in the ratio of 1:1. The LC−MALDI plate was analyzed on AB Sciex 4800 Plus TOF/TOF analyzer in reflector ion mode. The LC gradient for the separation of the peptides was made from two solvent systems. Solvent A contained 98% water, 2% ACN, and 0.1% TFA and solvent B contained 98% ACN, 2% water, and 0.1% TFA. Enzyme digested peptides were desalted with solvent A for 30 min on the Cap−trap C18 column (ABSciex, Foster City, CA, USA) with a flow rate of 20 µL/min in the reverse direction. After desalting, peptides were eluted from the column with 24 min gradient of solvent A and B, respectively, by using the following steps: 5% B for 2 min, 5−35% B for 20 min, 35–50% B for 24 min, 50–90% B for 26 min, 90% B for 30 min, 90–5% B for 32 min, and 5% B for 36 min. Most of the peptides were extracted between 10 min and 30 min. All of these peptides were spotted after every 7 s on the LC−MALDI plate with α−Cyano−4−hydroxycinnamic acid (CHCA). The flow rate of the solvent was 1.6 µL/min. The total run time was 36 min including equilibration.

### 2.6. MALDI TOF/TOF Analysis

A 4800 Plus MALDI TOF/TOF Analyzer (ABSciex, Foster City, CA, USA) was used for peptide mass fingerprinting (PMF) and MS/MS identification of peptides. The instrument was equipped with an Nd:YAG, 355 nm laser. The plate was calibrated using calmix, a mixture of six peptides, in the molecular weight range of 900–4000 Da. The spectra for the sample were recorded in the 500–4000 m/z range. For MS/MS analysis spectra were acquired with 2500 shots/spot. All of the MS/MS spectra were analyzed using Protein Pilot 2.0 software (ABSciex, Foster City, CA, USA) for sequence assignment and identification of mutation. The mutated Hb peptide fragments were validated further manually using MS and the MS/MS spectra of the individual peptide.

### 2.7. Database Generation and Searching

Single amino acid mutations were selected manually from the total entries in “A Database of Human Haemoglobin Variants and Thalassemia” included in the Globin Gene Server [28]. The database helped us by providing information about the nature of mutated amino acids and their position in the respective chain. Hb chain sequences, such as alpha, beta, gamma–1, gamma–2, and the delta chains were taken from NCBI and the mutations were incorporated in the respective sequences. The file was finally converted into fasta format and named Hb.fasta. The database was created with minimal and sophisticated information to save time and to specify the search using MS data. It contains the protein sequences of only single mutations in Hb. There are 1420 Hb variants entered in the globin gene server (till date 22 July 2022). Initial methionine was ignored in the positioning mutation. To identify the peptides, peptide masses obtained from the mass spectrometric analysis were searched using the Protein Pilot 2.0 software (ABSciex, Foster City, CA, USA) against the short database created by us. The detected protein threshold was fixed at a confidence score of 99.9%.

## 3. Results

### 3.1. Quality and Purity Assessment of Isolated Pure Hb and Recombinant Mutants

**Figure 1:**
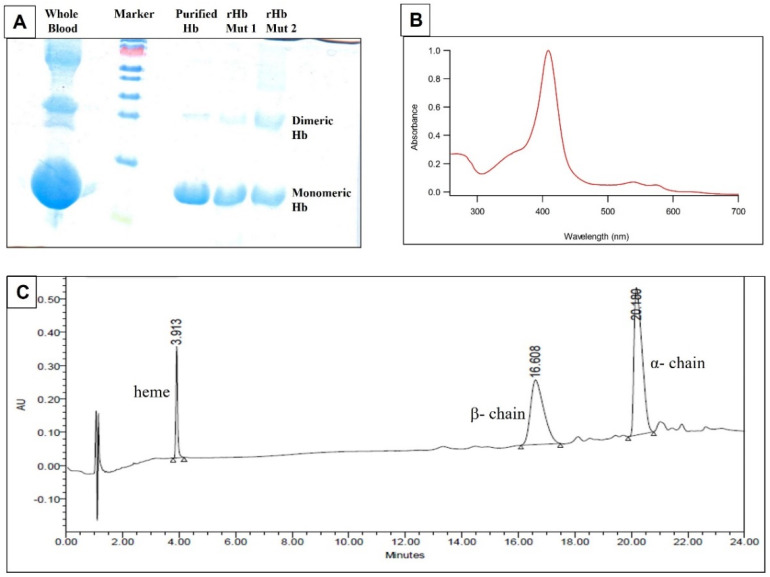
(**A**) SDS−PAGE: lane 1, whole blood; lane 2, marker; lane 3, purified hemoglobin (pure Hb) (isolated from hemoglobind matrix); lane 4, recombinant mutant 1; lane 5, recombinant mutant 2. (**B**) Representative absorbance spectrum of pure Hb. (**C**) Representative UPLC spectrum of pure Hb.

The experiments started with a purified protein. Hemoglobind matrix allows a single step isolation of pure Hb in minimal time with high recovery. Thus, Hemoglobind provides the easiest way to purify Hb from blood samples. Hb isolated from blood samples and the recombinant Hbs were found to be of high quality and purity as assessed by SDS−PAGE, absorbance, and UPLC (Figure 1). The spectral characteristics of recombinant Hb and Hb isolated from blood were found to be identical. Subsequently, the differences in the control and diseased Hbs were studied with mass spectrometry.

The purified Hb samples were analyzed by 15% SDS−PAGE, and they showed one monomeric hemoglobin band at approximately 16 kDa and one dimeric peak at approximately 32 kDa (Figure 1A). Other than these two bands, we did not observe any impurity of SDS−PAGE in our sample (Figure 1A). The absorbance spectrum of isolated Hb was collected in the wavelength range of 260–700 nm. Pure Hb exhibited spectra typical of globins with a characteristic Soret peak at ~411 nm and Q−bands in the region of 540–560 nm. The ratio of the absorbance value at Soret peak and at 280 nm was found to be 3.6 (Figure 1B). Usually, a ratio of 3.5 and higher indicates a high quality of Hb (Hoy et al., 2004). The UPLC chromatogram of isolated Hb also showed a pattern typical of a purified recombinant protein (Figure 1C). Purified Hb samples were separated on UPLC on the reverse phase C18 column. Heme gets resolved (retention time 3.931 min) and the two chains of beta and alpha are resolved at the retention time of 17.676 min and 20.470 min, respectively (Figure 1C). There were no other peaks observed, indicating the high quality of protein. The SDS–PAGE, absorbance, and UPLC results showed that all the Hb protein samples were pure and of high quality.

The use of Hemoglobind reduced the time to obtain pure Hb in an easy single–step procedure. Pure Hb protein is required specifically to optimize and standardize methods for diagnostics. Hemoglobind made Hb purification easier due to its high degree of specificity for Hb, binding up to 10 mg/mL of protein. For purification and/or analysis of Hb, a modest elevation in pH facilitated the desorption of Hb bound to Hemoglobind. The desorbed Hb was compatible with LC–MS, and other proteomics studies, as we verified, did not show any change in its intact mass either. This one−step affinity purification gave us the utmost purified Hb.

### 3.2. Low Sequence Coverage Was Obtained with the Conventional Method in a Single Spot

The single spot MS/MS analysis results showed sequence coverage to be too low, especially in alpha chains (Table S1), so there are chances of missed mutations if present in the unidentified sequence. The average sequence coverage for α−chain was 54.5% while for β−chain it was 81.4% with the single spot method.

### 3.3. Acetonitrile (10%) Treatment and LC−MALDI Improved the Sequence Coverage Significantly

We attempted to improve upon the sample processing to obtain higher sequence coverage. It was hypothesized that either the heme of Hb masked protease digestion process due to non−specific hydrophobic interaction with proteins, or that intact chains of Hb underwent precipitation and thus escaped protease digestion or that enough number of peptides were not extracted for significant MS signals in the solvent used. One solvent that could improve all the possibilities is ACN, and this solvent was used for MS analysis in literature before [16]. The average sequence coverage calculated with the ACN method for α chain was 65.3%, while for β chain it was 91.5%. The results with the ACN method for rHbs are shown in Table S2.

It was hypothesized at this point that the sequence coverage might improve if the trypsin digested peptides were first separated before spotting on MALDI plates. Liquid chromatography (LC) is one of the best methods for the separation of peptides before MS/MS analysis. LC−MALDI utilizes the separation power of liquid chromatography, thus allowing a larger separation of peptides to get them identified. Nano LC was used offline for the separation of peptides. The eluted samples from LC were spotted directly on the MALDI plate and subjected to MS/MS analysis. Typical LC chromatograms for some mutants are shown in Figure S1. Each of these standard steps were investigated for the sake of uniformity in output from a single laboratory for comparative purposes.

With 10% ACN treatment and the separation power of LC coupled with MALDI MS/MS, we got better separation of peptides resulting in improved sequence coverage, especially in the case of alpha chains (Table 1). The average sequence coverage for the α−chain was 77.8% while for the β−chain it was 90.0% (Table 1). From Table 1 it was evident that 10% ACN treatment, followed by LC separation of digested peptides, provided high quality MS/MS data. The sequence coverage obtained for the Hb mutants is summarized in Table 1. It is evident that sequence coverage is not 100% and needs further improvement. Though the sequence coverage improved, it was still not 100% and in some cases such as the rHb Mutant 2 (Table 1), the sequence coverage was still low and needed further improvement, especially for the α chain.

### 3.4. Digestion by Multiple Proteases Increased the Sequence Coverage up to 100%

Using a combination of ACN treatment and LC separation, MALDI TOF/TOF mass spectrometry yielded high sequence coverage for Hb chains. However, the sequence coverage was still not 100%, as per our objective. In order to achieve the goal, we explored other methods. So far, the protease digestion was performed by trypsin, the gold standard in MS. Trypsin digestion may lead to very small peptides that go undetected and missed cleavages could result in large peptides which are not sequenced properly. It was thus imperative to try other proteases as well for digestion. Here, we have compared the results for three proteases—trypsin, Glu−C, and Lys−C. In each case, the protocol we followed for MS and MS/MS analysis was the same as described in the previous section, which was the use of 10% ACN treatment prior to trypsin digestion, which was separated on LC and subjected to MALDI−TOF/TOF MS analysis. The results showing sequence coverage in rHbs are summarized in Table 2.

Trypsin cleaves after the C−terminal of lysine and arginine residues. However, it does not cleave if the proline is at the C−terminal of the cleavage site and skips at adjacent cleavage sites. Glu−C has maximal activity at either pH 4.0 or pH 8.0. At pH 4, it specifically cleaves at the C−terminal of glutamic acid while at pH 8.0 it cleaves the C−terminal of glutamic acid and additionally cleaves at the C−terminal of aspartic acid. Glu−C activity and cleavage specificity depend on pH and buffer conditions [29]. The source of Glu−C endoproteinase or V8 protease is *Staphyloccous aureus* and the source for Lys−C endopeptidase is *Lysobacter enzymogenesis*. Lys−C cleaves at the C terminus of lysine and its optimum working pH is 8.0. It is resistant to chemical denaturation and can sustain up to 8 M urea concentrations. These are all serine proteases and their active sites consist of the His, Asp, and Ser catalytic triad.

Table 2 depicts the sequence coverage in alpha and beta chains of rHb and its different mutants with trypsin, Glu−C, and Lys−C. Lys−C was found to be more effective in digestion as compared to trypsin, but mutations are not identified most of the time. Glu−C gave significantly less sequence coverage than trypsin. Trypsin digestion was able to identify the mutations, but sequence coverage was less. Thus, individually, none of the proteases could yield 100% sequence coverage, nor did they identify mutations consistently. It was then thought that if we combine the data obtained for individual proteases, the sequence coverage could improve.

When we combined the data from the three different enzymes, we found that the combination of trypsin and Glu−C is good at mutation identification but yields less sequence coverage (Table 3), especially in the α−chain in some cases. A combination of Glu−C and Lys−C maps all the peptides and hence yielded very good sequence coverage but was not able to identify the mutations consistently (Table 3). The combination of Lys−C and trypsin works best in providing good sequence coverage and identifying mutation (Table 3). Thus, combining MS/MS data obtained from two proteases, Lys−C and trypsin, helped in obtaining 100% sequence coverage for the Hb chain, a feat not yet reported in the literature. All mutations were also identified successfully using this strategy but sometimes we missed mutations; the method also became costly because of the use of multiple proteases. One α−chain mutant still showed ~92% sequence coverage, leaving scope for further improvement.

### 3.5. TFE Treatment Digestion Method Improved Sequence Coverage Significantly in rHb

By use of multiple proteases for Hb digestion and combining the data thereof, we achieved our target of 100% sequence coverage for Hb chains. However, such a protocol may not be cost−effective for diagnostic purposes. Using multiple proteases would add to the cost, time, and labor—all factors that were against a diagnostic procedure being clinically viable. Such a protocol could be used if the patient condition is deleterious and it is extremely important to know the precise mutation for the design of a therapeutic intervention. Hence, we resorted back to improving sample processing by simpler methods so that we can achieve near 100% sequence coverage while also identifying the single amino acid mutation.

**Figure 2:**
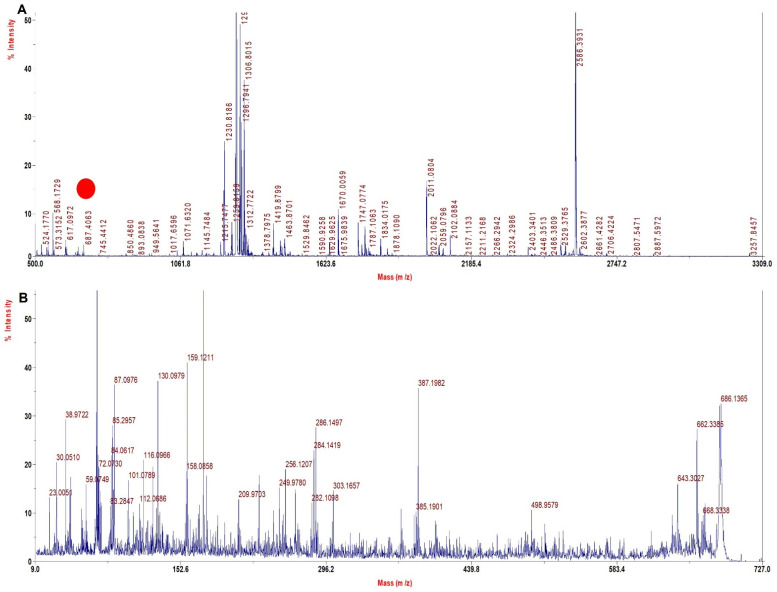
MALDI−MS and MALDI MS/MS spectra for the signature peptide from Mutant 1: Beta 63 (E7) His > Gly (**A**). PMF spectrum showing signature peptide obtained by TFE treated in−solution tryptic digestion. (**B**) MALDI−MS/MS spectrum of the signature peptide 687.4 m/z of mutant 1.

**Figure 3:**
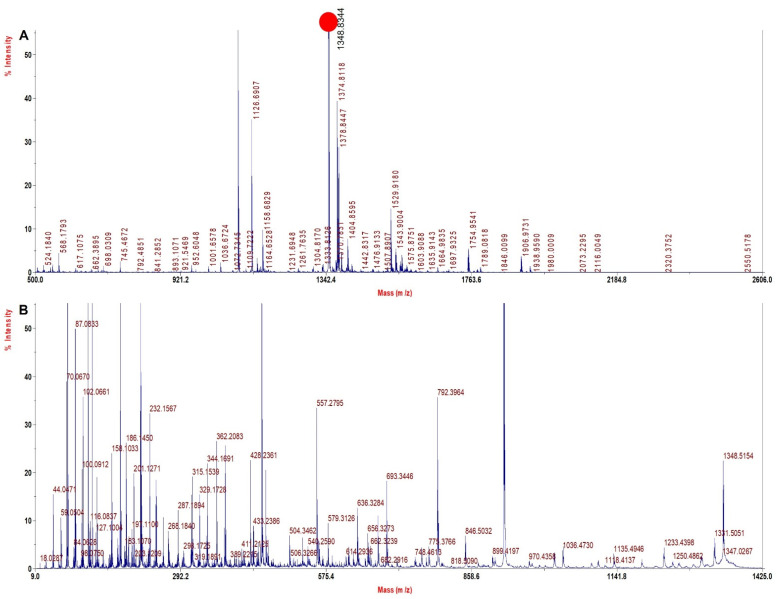
MALDI−MS and MALDI MS/MS spectra for the signature peptide from Mutant 2: Beta 28 (B10) Leu > Phe (**A**). PMF spectrum showing signature peptide obtained by TFE treated in−solution tryptic digestion. (**B**) MALDI−MS/MS spectrum of the signature peptide 1348.4 m/z of mutant 2.

**Figure 4:**
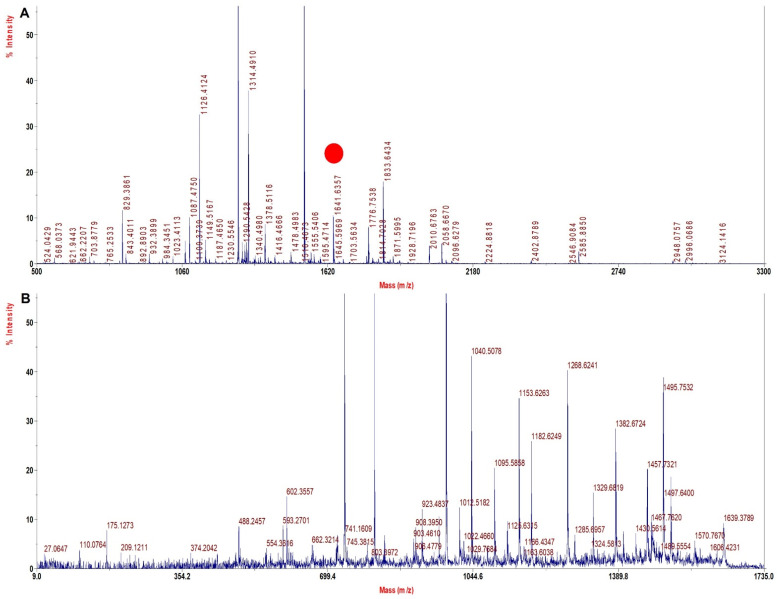
MALDI−MS and MALDI MS/MS spectra for the signature peptide from Mutant 3: Beta 67 (E11) Val > Ala (**A**). PMF spectrum showing signature peptide obtained by TFE treated in−solution tryptic digestion. (**B**) MALDI−MS/MS spectrum of the signature peptide of 1641.6 m/z.

**Figure 5:**
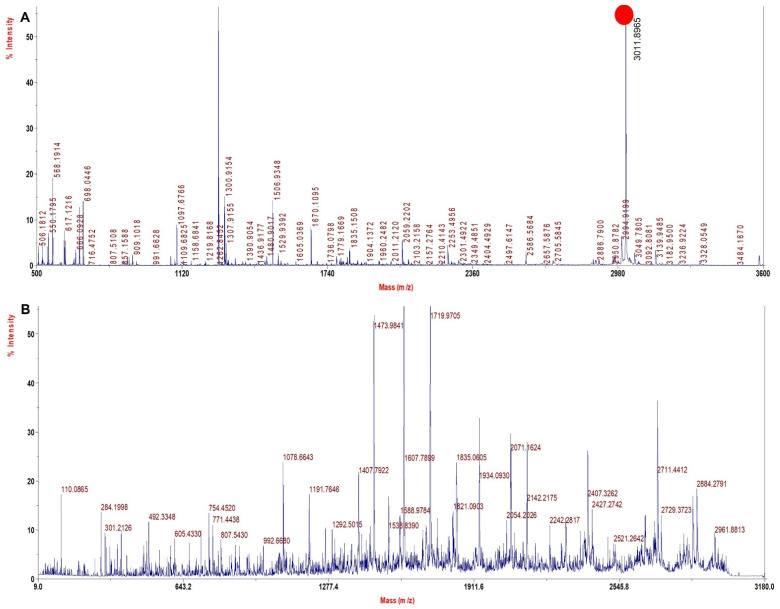
MALDI−MS and MALDI MS/MS spectra for the signature peptide from Mutant 4: Alpha 62 (E11) Val > Asn (**A**). PMF spectrum showing signature peptide obtained by TFE treated in−solution tryptic digestion. (**B**) MALDI−MS/MS spectrum of the signature peptide of 3011.5 m/z.

A literature search and our experience with several solvents explored for sample processing indicated TFE to be a viable solvent that may improve sequence coverage. TFE helps in the removal of heme and also helps in the solubilization of both intact Hb chains and peptides, thus improving digestion and the extraction of digested peptides. Since with the TFE method the sample processing resulted in high sequence coverage for rHb and its mutants (Table 4) with high quality of MS spectra (Figure 2, Figure 3, Figure 4 and Figure 5) and accurate identification of mutations, this method was further adopted for the investigation of Hb variants from patient samples.

Figure 2A shows MS spectra for Mutant 1 (β 63 (E7) His > Gly) depicting the signature tryptic peptide V^60^KAGGKK^66^ of monoisotopic m/z of 687.9 Da. In β tryptic digested peptide, the difference between normal and mutated one is 80 Dalton, which suggests that histidine is replaced by glycine (His^63^→Gly^63^). The MS/MS spectra of signature tryptic peptide VKAGGKK is shown in Figure 2B. The sequence coverage obtained from the MS/MS spectra of all such peptides was 97.9% in α and 100% in β chains with our database defined search in ProteinPilot software.

Figure 3A shows the MS spectra for Mutant 2 (β 28 (B10) Leu > Phe) depicting the signature tryptic peptide V^18^NVDEVGGEAFGR^30^ of monoisotopic m/z of 1348.4 Da. In β tryptic digested peptide, the difference between the normal and the mutated one is 34 Dalton, which suggests that leucine is replaced by phenylalanine (Leu^28^→Phe^28^). The MS/MS spectra of signature tryptic peptide VNVDEVGGEAFGR is shown in Figure 3B. The sequence coverage finally obtained for Hb was 99.3% in α chains and 99.3% in β with our database defined search in ProteinPilot software.

Figure 4A shows the MS spectra for Mutant 3 (β 67 (E11) Val > Ala) depicting signature tryptic peptide A^67^LGAFSDGLAHLDNLK^82^ of monoisotopic m/z of 1641.6 Da. In β tryptic digested peptide, the difference between the normal and the mutated one is 28 Dalton, which suggests that valine is replaced by alanine (Val^67^→Ala^67^). The MS/MS spectra of signature tryptic peptide ALGAFSDGLAHLDNLK is shown in Figure 4B. The sequence coverage finally obtained for Hb was 97.3% in α chains and 99.3% in β with our database defined search in ProteinPilot software.

Figure 5A shows MS spectra for Mutant 4 (α 62 (E11) Val > Asn) depicting the signature tryptic peptide of N^62^ADALTNAVAHVDDMNALSALSDLHAHK^89^ monoisotopic m/z of 3011.5 Da. In β tryptic digested peptide, the difference between the normal and the mutated one is 15 Da, which suggests that valine is replaced by alanine (Val^62^→Asn^62^). The MS/MS spectra of signature tryptic peptide NADALTNAVAHVDDMPNALSALSDLHAHK is shown in Figure 5B. The sequence coverage finally obtained for Hb was 99.3% in α−chains and 99.3% in β−chains with our database defined search in ProteinPilot software.

### 3.6. TFE Modified Digestion Method Improved Sequence Coverage Significantly in Human Hb Variants

**Figure 6:**
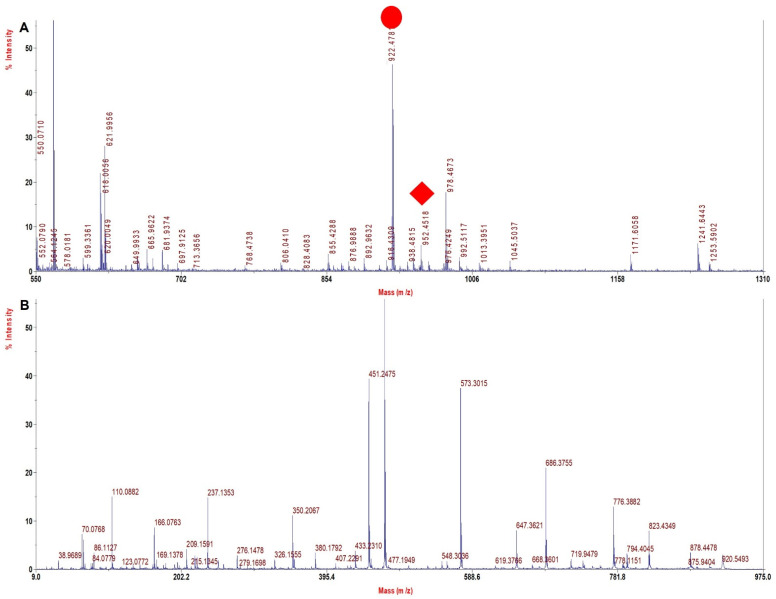
MALDI−MS and MALDI MS/MS spectra for the signature peptide from HbS heterozygous (1908) Beta 6 (A3) Glu > Val (**A**). PMF spectrum showing signature peptide obtained by TFE treated in−solution tryptic digestion. (**B**) MALDI−MS/MS spectrum of the signature peptide 922.4 m/z of HbS heterozygous sample.

**Figure 7:**
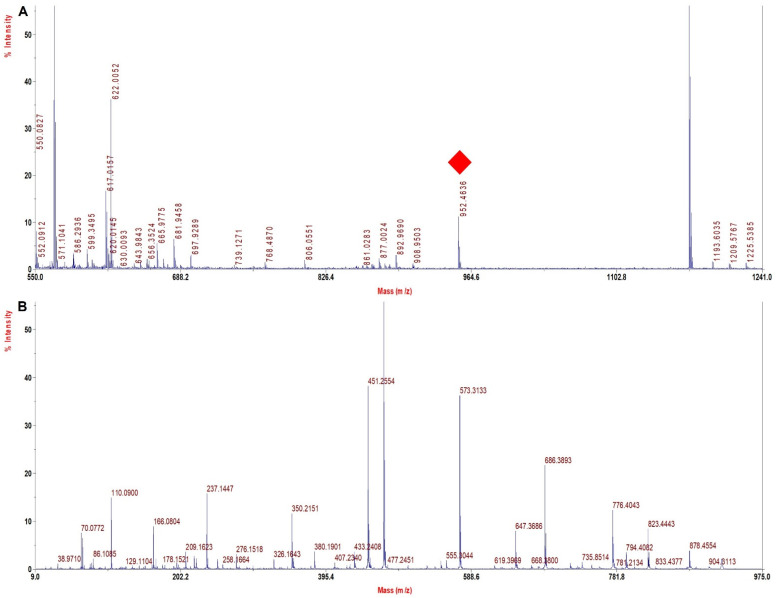
MALDI−MS and MALDI MS/MS spectra for the signature peptide from HbS homozygous (1909) Beta 6 (A3) Glu > Val (**A**). PMF spectrum showing signature peptide obtained by TFE treated in−solution tryptic digestion. (**B**). MALDI−MS/MS spectrum of the signature peptide 922.4 m/z of HbS homozygous sample.

**Figure 8:**
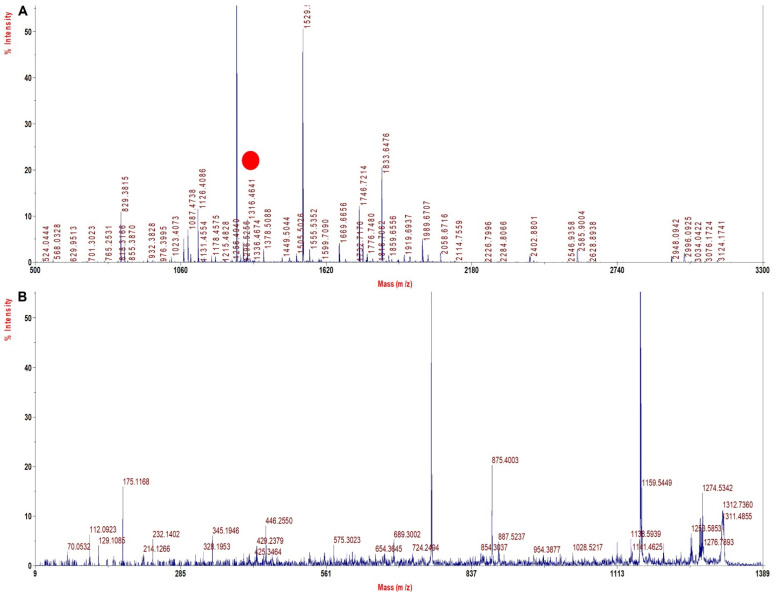
MALDI−MS and MALDI MS/MS spectra for the signature peptide from HbE (1963) Beta 26 (B8) Glu > Lys (**A**). PMF spectrum showing signature peptide obtained by TFE treated in−solution tryptic digestion. (**B**). MALDI−MS/MS spectrum of the signature peptide 1316.5 m/z of HbE sample.

Representative MALDI−MS and MALDI−MS/MS spectra for signature peptides of Hb variants from patient samples are shown in Figure 6, Figure 7 and Figure 8. The spectral quality was good and reproducible.

Figure 6A depicts the MS spectrum of HbS heterozygous β 6 (A3) Glu > Val showing the tryptic peptide V^1^HLTPVEK^8^ of monoisotopic m/z of 922.47. The MS/MS spectrum of the signature tryptic peptide VHLTPVEK is shown in Figure 6B. In β tryptic digested peptide VHLTPVEK of the HbS heterozygous sample, the difference between y (3−2 ion) in the normal and the mutated one was 30 Da, which indicated that glutamic acid was replaced by valine (Glu^6^→Val^6^). The sequence coverage obtained from detailed MS/MS analysis was 96.6% in α chains and 100% in β chains with our database defined search in ProteinPilot software (Table 4).

Figure 7A depicts the MS spectrum of HbS homozygous β 6 (A3) Glu > Val showing the tryptic peptide V^1^HLTPVEK^8^ of monoisotopic m/z of 922.47. The MS/MS spectrum of the signature tryptic peptide VHLTPVEK is shown in Figure 7B. In β tryptic digested peptide VHLTPVEK of the HbS heterozygous sample, the difference between y (3−2 ion) in the normal and the mutated one was 30 Dalton, which indicated that glutamic acid was replaced by valine (Glu^6^→Val^6^). The sequence coverage obtained from detailed MS/MS analysis was 70.4% in α chains and 100% in β chains with our database defined search in ProteinPilot software (Table 4).

Figure 8A depicts the MS spectrum of HbE β 26 (B8) Glu > Lys showing the tryptic peptide V^1^NVDEVGGKALGR^13^ of monoisotopic m/z of 1316.46. The MS/MS spectrum of the signature tryptic peptide VNVDEVGGKALGR is shown in Figure 8B. In β tryptic digested peptide VNVDEVGGKALGR of HbE sample, the difference between y (5−4 ion) in the normal and in the mutated one was 1 Dalton, indicating that glutamic acid was replaced by lysine (Glu^26^→Lys^26^). The sequence coverage deduced was 99.3% in α chains and 100% in the β chain with our database defined search in ProteinPilot software. It is thus evident that all the Hb variants investigated here with TFE method sample processing yielded near 100% sequence coverage (Table 4), especially for the β chain. The sequence coverage was low for α chains in only one case, but since most common variants are in the β chain, the method is still acceptable. The mean for sequence coverage in β chains turned out to be 100%, while it was 95% for α chains, which is extremely significant, and no other group has reported this earlier (Table 4). It can thus be anticipated that this method will be capable of identifying most variants with almost complete sequence coverage in both α and β chains. This method was also sensitive and able to resolve peptides with <1 Da of difference.

Since the sample processing with the TFE method, followed by LC separation and the MALDI−MS and MALDI−MS/MS spectral analysis of the Hb chain seems to have the potential to be a diagnostic tool to identify Hb disorders, some of the essential features and parameters of signature peptides of known mutants and variants are listed in Table 5. Table 5 summarizes the precursor ion mass for both wild type and mutated peptides or rHbs and human Hb variants. It also shows the signature tryptic peptides obtained after digestion. The difference in mass among the wild type and mutated peptide and the sequencing results of signature peptides infers the mutation. This strategy can be applied to all known variants of Hb for the identification of mutations. For new mutants of Hb, the sequence obtained from MS/MS analysis will have to be interpreted.

## 4. Discussion

In our study, we primarily optimized and tested a sample processing method for Hb variant identification. Initially, we tried various sample processing methods like acetonitrile treatment, digestion with multiple proteases, and TFE treated trypsin digestion in recombinant hemoglobin samples, and we found that the TFE method is the most suitable method for the identification of a single mutation in the globin chains of rHbs. We further tested this method for the analysis of Hb variants.

The routine/conventional techniques used widely in the field of clinical diagnosis for Hb disorders have their own limitations leading to the ambiguity of precise detection of Hb mutants in patient samples. MS emerged as a powerful diagnostic tool in the identification of Hb variants in the last three decades [10,11,12]. Two different approaches—top−down [30,31] and bottom−up, were generally used to characterize and identify Hb variants by MS [32]. In the top−down proteomics approach, intact protein samples are subjected to fragmentation and tandem MS analysis is performed for acquiring protein sequence coverage, whereas in the bottom−up proteomics approach, proteins are digested with enzymes to form peptides and then these peptides are further subjected to MS and tandem MS for obtaining protein sequence coverage. These peptide sequences are further searched in an online database to identify protein sequences. In the case of top−down MS analysis, commonly used activation modes are electron capture dissociation (ECD) and electron transfer dissociation (ETD) [33,34]. These two activation modes permit a more efficient fragmentation which results in the production of product ions. The top−down approach does not require any sample preparation and hence saves time [35]. Cooper and co−workers demonstrated the detection of the Hb variant by direct surface sampling with top−down high−resolution MS in newborns [36]. A top−down electron transfer dissociation mass spectrometry method was developed for Hb β−chain analysis against the product ion list [37]. Bottom−up approaches give precise and high throughput identifications with the targeted screening of common Hb variants, e.g., HbS, HbC, and HbE [38,39]. However, targeted peptide measurements do not allow the identification of uncommon Hb mutations or new mutants if they are not present in the targeted Hb variant list. Hence, methods still need to be developed to identify variants without using a targeted approach, such that new mutations can be identified. Mandal and his group showed the identification of HbE, HbQ, and HbD−punjab in a data−independent approach [12] using peptide based identification.

There is a previous study which showed the evidence of use of the bottom−up approach for such Hb variant identification, especially the use of the targeted−proteomics approach [30]. In this approach, the signature peptide of each variant was targeted for identification by the PMF−based method, often followed by further sequencing of the signature peptide [30]. Also, if the mutation is known then the product ion list could be incorporated in the MS/MS list and only those peptides if present were fragmented [40]. A bottom−up study was performed through nano−LC to study Hb variant analysis using the data independent approach with digestion with multiple enzymes, and Hb variant databases (HbVDTryp, HbVDChymotryp, and HbVDGluC) were created [12]. We have used the bottom−up approach in our study, and our main objective was to use MS/MS sequence analysis of all peptides of Hb chains to get an amino acid sequence coverage of 100%, which is an experimental challenge. The ability to obtain 100% sequence coverage of Hb chains will allow the identification of new Hb variants as well as the identification of any variant in a model−free or untargeted approach. Though getting 100% sequence coverage of the protein sequence is a prerequisite for the data independent approach (DIA) but such success has not been reported to this point. A protein sequence database was thus generated from the information obtained from the globin gene server [28]. The fasta sequence of alpha, beta, gamma, and delta chains were obtained from the NCBI database and introduced into our database with appropriate substitutions of amino acids. The mutations having two or more substitutions were left out and for all others, the mutations were manually incorporated into the database. All of the results were searched against this database, which made the search easy and fast.

In−solution digestion of a pure protein sample increases the number of peptides available for analysis [41]. Also, using in−solution digestion was beneficial since it saved time in running gels and then performing in−gel digestion on excised protein bands. From a diagnostic viewpoint, this would save both time and cost. Modification of the conventional methods of digestion where denaturants like urea [42,43,44], GdnHCl [44], and ACN [45,46] are used could help improve sequence coverage during peptide identification, but the use of chaotropic agents adds an extra cleanup step as well. We tried to keep sample processing to a minimum by using pure Hbs. With our aim of obtaining perfect sequence coverage, we tried different strategies such as acetonitrile treatment, nano−LC separation of peptides, the use of multiple proteases for digestion and combination of results obtained thereby, and a TFE−based sample preparation for MALDI−MS/MS analysis. In each case, we saw progressive improvement in MS/MS spectral data, sequence coverage and identification of mutation. However, the best result was obtained when MS/MS analysis was performed individually after digestion by trypsin, Glu−C, and Lys−C, and the data were combined in groups. The sequence coverage was 100%, a feat not reported in the literature previously. We found that when the MALDI results for trypsin and Lys−C were combined by mapping the peptide in order, 100% sequence coverage was obtained along with the mutations being identified with ease.

Though the combination of data obtained by the digestion of different proteases resulted in 100% sequence coverage, this strategy is cumbersome, complex and would be time−consuming and costly for diagnostic success. Hence, we looked for alternatives and the results obtained upon TFE based sample processing stood out, thus digestion by multiple proteases was not necessary.

TFE treated trypsin digestion coupled with LC−MALDI was successfully optimized to overcome the problem of sequence coverage and in the identification of the single mutation in hemoglobin variants. TFE probably denatured and solubilized the protein chains completely, thus giving complete sequence coverage and improving the quality of MS/MS spectra, helping in mutation identification [47]. Moreover, it is extremely helpful for hydrophobic proteins, e.g., sickle cell hemoglobin, which is harder to solubilize. Hence, TFE improved protein digestion results in 100% sequence coverage, especially in the β chain. TFE digestion protocols provide better sample recovery and are reproducible. The method thus provided a solution for a long−term problem in the inability of getting complete sequence coverage for Hb chains. Improved sequence coverage enables improved protein identification. This strategy reduces the running time and diagnosis costs and may be developed into a diagnostic tool in the future.

**Limitations:** There are some limitations in our study: First, we have not optimized this TFE digestion method for double mutants of Hb variants/ recombinant Hb samples. Second, it would be more appropriate if we were able to test Hb variants with 10% acetonitrile treatment as well as with multiple proteases such as Trypsin, Glu−C, and Lys−C. However, due to the low volume of the blood samples, we have only tested the TFE method. Third, there is still a chance of improvement which could give us 100% sequence coverage in both chains of hemoglobin. Fourth, we have not tested rHb samples and Hb variants with a more sensitive MS instrument such as the Orbitrap (Thermo Fisher Scientific). In the future, we can identify our peptides with Orbitrap for greater sequence coverage. Fifth, In the future, this TFE based method could be applied to other Hb variants for identification and greater sequence coverage. Sixth, we have not tried another matrix, which is also a limitation of our study. However, in the future, we can plan the study with various matrices for sequence improvement in Hb variants.

## 5. Conclusions

A sensitive, robust, and reproducible method was thus developed to identify/characterize single substitution mutations in the Hb disorder variants which require a very small amount of pure sample. It simply used pure Hb isolated from blood in a single step by using the Hemoglobind matrix, followed by TFE based sample processing and digestion by trypsin, followed by nano−LC separation. The separated peptides were spotted onto MALDI plates upon elution from a nano−LC column and subjected to MALDI MS and MS/MS analysis. The strategy resulted in 100% sequence coverage in the β−chain. The method was tested with recombinant Hb mutants as a proof of concept. The method was finally tested with three clinical hemoglobin variant (HbS heterozygous, HbS homozygous, and HbE) samples. A hemoglobin variant protein sequence database was also created to specify the search and reduce the search time. The main highlight of this method is its non−targeted approach for the identification of Hb variant and its reproducibility to yield 100% sequence coverage for the β−chain with precise identification of all the mutations. This method has the potential to become a regular screening/diagnosis tool for the identification of single amino acid mutations in Hb variants.

## Figures and Tables

**Table 1:** Sequence coverage for 10% acetonitrile treated recombinant Hbs (rHbs) in LC−MALDI.

Sample Name	Sequence Coverage (%)	
	Alpha	Beta
rHb Control	88.0	96.6
rHb Mut 1 β 63 (H > G)	82.4	97.3
rHb Mut 2 β 28 (L > F)	70.4	93.9
rHb Mut 3 β 67 (V > A)	69.0	79.6
rHb Mut 4 α 62 (V > N)	79.6	82.3

**Table 2:** Sequence coverage for recombinant Hbs with 10% acetonitrile treated Hbs digested with three different enzymes in LC−MALDI.

Mutation Name	Sequence Coverage LC−MALDI (ACN) (%)						Mutation Identified Trypsin/Glu−C/Lys−C
	Trypsin		Glu−C		Lys−C		
	Alpha	Beta	Alpha	Beta	Alpha	Beta	
rHb Control	89.4	99.3	31.7	91.2	96.5	93.9	
rHb Mut 1 β 63 (H > G)	85.9	100	85.9	100	91.5	95.9	√/−/−
rHb Mut 2 β 28 (L > F)	54.9	86.4	74.6	100	96.5	98.6	√/√/−
rHb Mut 3 β 67 (V > A)	69.0	79.6	92.3	87.8	90.8	98.6	√/−/−
rHb Mut 4 α 62 (V > N)	79.6	82.3	66.2	85.7	96.5	95.9	√/−/−
Human Hb Control	99.3	99.3	76.8	99.3	88.7	98.0	

**Table 3:** Sequence coverage in rHbs with 10% acetonitrile treated Hbs digested with three different enzymes in combination in LC−MALDI.

Sample Name	Sequence Coverage (%)					
	Trypsin + Glu−C Combined		Glu−C + Lys−C Combined		Lys−C + Trypsin Combined	
Chain	α	β	α	β	α	β
Human Hb Control	100	100	100	100	100	100
rHb Control	80.3	96.6	96.5	97.3	100	95.2
rHb Mutant 1	94.4	100	100	100	91.5	97.3
rHb Mutant 2	93.0	100	100	100	100	100
rHb Mutant 3	92.3	100	100	100	100	100
rHb Mutant 4	88.7	100	100	100	100	100

**Table 4:** Sequence coverage for rHbs and human Hb variants with TFE mediated digestion in LC coupled with MALDI MS/MS.

Mutation Name	Sequence Coverage (%)		Mutation Identified
	Alpha	Beta	
rHb Control	97.3	99.3	−
rHb Mut 1 β 63 (H > G)	97.9	100	√
rHb Mut 2 β 28 (L > F)	99.3	99.3	√
rHb Mut 3 β 67 (V > A)	97.9	99.3	√
rHb Mut 4 α 62 (V > N)	99.3	99.3	√
Human Hb Control	99.3	99.3	−
HbS Heterozygous β 6 (E > V) (1908)	96.6	100	√
HbS Homozygous β 6 (E > V) (1909)	70.4	100	√
HbE β 26 (E > K) (1963)	99.3	100	√

**Table 5:** Summarized mass spectrometric data for rHb mutants and Hb variants.

Sample	Wild Type Precursor Ion MH^+^	Mutated Precursor Ion MH^+^	Mass Difference (Dalton)	Tryptic Signature Peptide	Inference
rHb Mut 1	765.2	687.9	−80	VKAGGKK	β 63 His > Gly
rHb Mut 2	1314.4	1348.4	+34	VNVDEVGGEAFGR	β 28 Leu > Phe
rHb Mut 3	1669.6	1641.6	−28	ALGAFSDGLAHLDNLK	β 67 Val > Ala
rHb Mut 4	2995.5	3010.5	+15	NADALTNAVAHVDDMPNALSALSDLHAHK	α 62 Val > Asn
HbS hetero	952.4	922.4	−30	VHLTPVEK	β 6 Glu > Val
HbS homo	952.4	922.4	−30	VHLTPVEK	β 6 Glu > Val
HbE	1316.4	1315.5	−1	VNVDEVGGKALGR	β 26 Glu > Lys

## Data Availability

Not applicable.

## Supplementary Materials

The following supporting information can be downloaded at: www.mdpi.com/1420-3049/27/19/6357/s1, Supplementary Information File S1: Figure S1: LC chromatogram for trypsin digested rHb control and mutants and Human Hb. (A) Human Hb control (B) rHb Control (C) Mutant 1: Beta 63 (E7) His > Gly (D) Mutant 2: Beta 28 (B10) Leu > Phe (E) Mutant 3: Beta 67 (E11) Val > Ala (F) Mutant 4: Alpha 62 (E11) Val > Asn. Table S1. Sequence coverage for recombinant Hbs in single spot MS/MS analysis. Table S2. Sequence coverage for recombinant Hbs upon 10% acetonitrile treatment. Supplementary Information File S2: Hb variant database.

## Abbreviations

α: alpha; β: beta; ABC: Ammonium bicarbonate; ACN: Acetonitrile; FA: Formic acid; Glu−C: Glu−C Endoproteinase; Hb: Hemoglobin; LC: Liquid chromatography; Lys−C: Lys−C Endoproteinase; MS: Mass Spectrometry; MALDI TOF/TOF: Matrix Assisted Laser Desorption Ionization Time of Flight/Time of Flight; rHb: Recombinant hemoglobin; TFA: Trifluoroaceticacid, TFE: 2, 2, 2−Trifluoroethanol.
