# Supplementary material for: Optimization and Identification of Single Mutation in Hemoglobin Variants with 2,2,2 Trifluoroethanol Modified Digestion Method and Nano−LC Coupled MALDI MS/MS
Source: Molecules. 2022 Sep 26;27(19):6357. doi: 10.3390/molecules27196357 (PMC9572498; doi:10.3390/molecules27196357)
Supplement: Supplementary file 1 [file molecules-27-06357-s001.zip › molecules-1877166-supplementary.pdf]

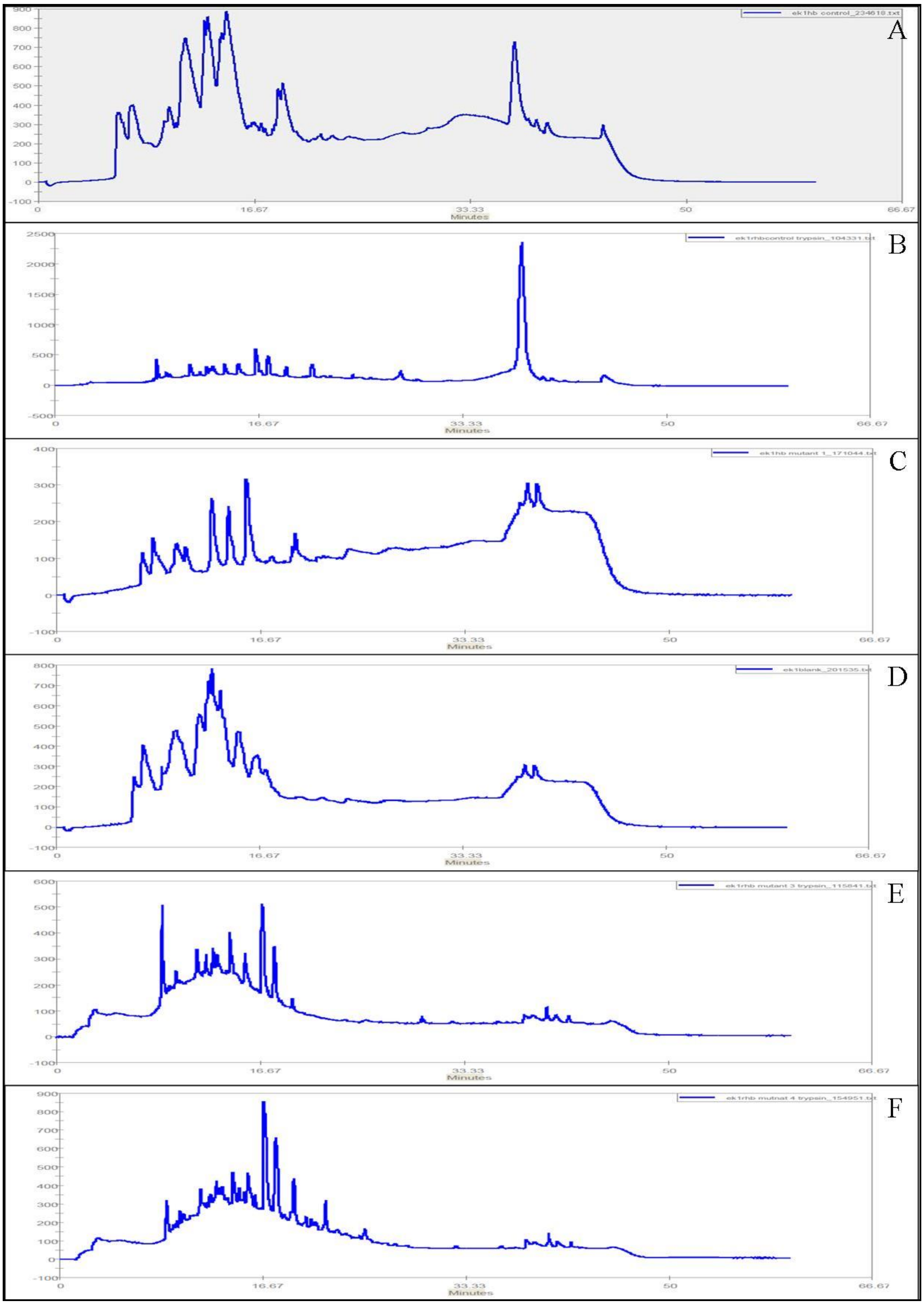

Figure S1. LC chromatogram for trypsin digested rHb control and mutants and Human Hb (A). Human Hb control (B). rHb Control (C). Mutant 1: Beta 63 (E7) His > Gly (D). Mutant 2: Beta 28 (B10) Leu > Phe (E). Mutant 3: Beta 67 (E11) Val > Ala (F). Mutant 4: Alpha 62 (E11) Val > Asn.

Table S1. Sequence coverage for recombinant Hbs in single spot MS/MS analysis.

**Sample Name**	**Sequence Coverage (%)**	
	Alpha	Beta
**rHb Control**	55.6	85.2
**rHb Mut 1 β 63 (H>G)**	64.1	70.9
**rHb Mut 2 β 28 (L>F)**	43.0	88.4
**rHb Mut 3 β 67 (V>A)**	54.9	79.2
**rHb Mut 4 α 62 (V>N)**	54.9	83.2

Table S2. Sequence coverage for recombinant Hbs upon 10% acetonitrile treatment.

**Sample Name**	**Sequence Coverage (%)**	
	Alpha	Beta
rHb Control	63.4	93.9
rHb Mut 1 β 63 (H>G)	62.0	96.6
rHb Mut 2 β 28 (L>F)	38.7	91.2
rHb Mut 3 β 67 (V>A)	83.1	87.8
rHb Mut 4 α 62 (V>N)	79.6	88.4
